# Isolation and characterization of *Mycobacterium bovis *strains from indigenous Zambian cattle using Spacer oligonucleotide typing technique

**DOI:** 10.1186/1471-2180-9-144

**Published:** 2009-07-20

**Authors:** Musso Munyeme, Leen Rigouts, Isdore Chola Shamputa, John Bwalya Muma, Morten Tryland, Eystein Skjerve, Berit Djønne

**Affiliations:** 1 Department of Disease Control, University of Zambia, School of Veterinary Medicine, PO Box 32379 Lusaka, Zambia; 2 Mycobacteriology Unit, Prince Leopold Institute of Tropical Medicine, 155 National Straat, 200 Antwerp, Belgium; 3 Department of Food Safety and Infection Biology, Section of Arctic Veterinary Medicine, Norwegian School of Veterinary Science, Stakkevollveien 23, N-9010 Tromsø, Norway; 4 Department of Food Safety and Infection Biology, Norwegian School of Veterinary Science, PO Box 8146 Dep, 0033 Oslo, Norway; 5 Department of Animal Health, National Veterinary Institute, PO Box 750 Dep, 0033 Oslo, Norway; 6 Tuberculosis Research Section, National Institutes of Health, LCID/NIAID, Bethesda, MD 20892, USA

## Abstract

**Background:**

Bovine tuberculosis (BTB), caused by *Mycobacterium bovis*, has remained a major source of concern to public health officials in Zambia. Previous investigations have used traditional epidemiological methods that are unable to identify the causative agent and from which dynamics of disease dispersion is difficult to discern. The objective of this study was to isolate, characterize and determine the genetic diversity and relatedness of *M. bovis *from major cattle rearing districts in Zambia by spoligotyping. A total of 695 carcasses were examined and 98 tissues had gross post-mortem lesions compatible with BTB.

**Results:**

Forty-two out of the ninety-eight suspected tissues examined had culture properties characteristic of mycobacteria from which 31 isolates yielded interpretable spoligotypes. This technique showed good discriminatory power (HGDI = 0.98), revealing 10 different spoligotype patterns. Twenty-seven isolates belonged to one cluster with more than 95% similarity and inside the cluster, one predominant spoligotype was found in 20 (64.5%) of the isolates tested. The highest number of spoligotypes was observed among samples from Namwala district. Spoligotypes from 26 (83.9%) of the isolates belonged to five spoligotypes that have been reported before while the remaining 5 (16.1%) isolates had unique spoligotypes that are being reported for the first time; these have been assigned numbers SB1763 to SB1767. Five of the 6 districts had the predominant spoligotype (SB0120).

**Conclusion:**

The study has described the dispersion patterns of *M. bovis *in Zambian cattle for the first time and has identified 5 spoligotype patterns specific to Zambia. The observation of an overlap in the spoligotype pattern SB0120 in 5 of the 6 districts suggests the probability of sharing a common source of infection.

## Background

Bovine tuberculosis (BTB), caused by *Mycobacterium bovis*, has been reported to be endemic in the Zambian traditional livestock sector [1-3], with relatively high prevalence being recorded in areas within and adjacent the Kafue Basin [1,4,5]. Prevalence rates at individual animal level vary from 0.8% in low prevalence settings to 9.6% in high prevalence settings, whilst herd level prevalence vary from 5.6% in low prevalence settings to 49.8% in high prevalence settings [3,4]. Recent survey data suggest that areas of high prevalence settings exist within the country [3]. One such area being the Kafue Basin of Zambia, were the livestock/wildlife interface forms a unique risk platform in terms of spread of infectious diseases among animals (both domestic and wild) [4-6].

BTB is one of the most common abattoir findings during meat inspection and a significant reason for organ condemnation [7,8]. The lack of abattoirs in most districts, coupled with the high cost of mechanized transport, entails cattle travelling long distances "on the hoof", sometimes passing through two or more districts before reaching the abattoirs. This kind of animal movement has been identified as the major hindrance in the control of most economically important diseases of livestock in Zambia [9]. Similarly, strains of *Mycobacterium bovis *may be spread across districts due to these uncontrolled animal movements. However, there is no information with regards to the molecular epidemiology of BTB in Zambia.

Molecular typing techniques have contributed greatly to the knowledge of inter-bovine and interspecies transmission of bovine tuberculosis [10-13]. The most widely used DNA typing techniques for *M. bovis *include IS*6110 *in restriction fragment length polymorphism (RFLP) typing [14], spacer oligonucleotide typing (Spoligotyping) [15] and variable number of tandem repeat (VNTR) typing [14-16]. RFLP is less desirable because it requires large amounts of DNA, is not based on Polymerase Chain Reaction (PCR), is time consuming, and poorly resolve strains of *M. bovis *owing to low copy numbers of IS*6110 *elements [17]. Both VNTR and Spoligotyping are PCR based, easy to perform, require little amounts of DNA, and can be used even with non-viable organisms. Spoligotyping has been more widely applied in part because it is fast and more importantly the technique can simultaneously detect and differentiate *M. bovis *from *M. tuberculosis *strains [15,16,18,19]. In addition, Spoligotyping patterns can be easily compared with results from other countries by use of a freely accessible international data base [20].

The objective of this study was to determine the genetic diversity and relatedness of BTB isolates from cattle in Zambia.

## Results

**Figure 1:**
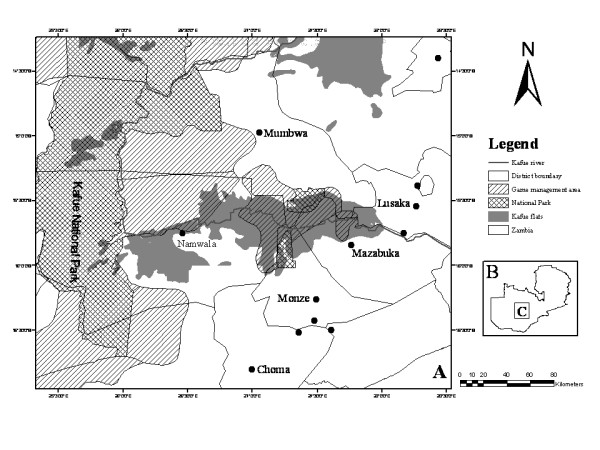
**Map of the Kafue Basin**. A – indicates major districts. B – insert of map of Zambia. C – study area.

Out of the 695 carcasses examined, 98 (14.1%) tissues and organs from the carcasses had gross characteristic lesions suggestive of tuberculous lesions. When subjected to culture on pyruvate enriched Lowenstein Jensen media, only 42 (6%) of the tissues resulted in discernable colony growth with properties suggestive of mycobacteria but only 33 (4.7%) samples were acid-fast positive by smear microscopy. Out of this number, 31 isolates yielded interpretable spoligotypes of *M. bovis *with all the six major districts around the Kafue Basin contributing at least one isolate each; Namwala (*n *= 12), Lusaka (*n *= 6), Mumbwa (*n *= 5), Monze (*n *= 5), Mazabuka (*n *= 2), Choma (*n *= 1) (Figure 1 and Table 1). All isolates lacked the spacers 3, 9, 16 and also from 39 to 43, a characteristic feature that distinguishes *M. bovis *from *M. tuberculosis *[15].

**Table 1:** Distribution of spoligotypes of *Mycobacterium bovis *isolates from cattle in six different districts of Zambia in 2004

	DISTRIBUTION OF SPOLIGOTYPES PER DISTRICT								
Isolate	Spoligotype	L	M	C	M	M	N	Total	Frequency
	SB Number*	S	Z	H	B	Z	M	**No**.	(%)
		K	K	M	W	E	A		
C9	**SB1767**					1		1	3.2
C19	**SB0162**						1	1	3.2
C21	**SB1763**				1			1	3.2
C26	**SB1764**						1	1	3.2
C14	**SB1572**	1						1	3.2
C42	**SB1765**						1	1	3.2
C16	**SB1536**						1	1	3.2
C4, C13, C15	**SB0871**			1	1		1	3	9.7
C41	**SB1766**						1	1	3.2
C2, C3, C5,									
C6, C8, C17,									
C18, C22,									
C24, C25,									
C27, C28,	**SB0120**	5	2		3	4	6	20	64.5
C29, C31,									
C38, C39,									
C40, C44,									
C45, C46									

**Total number**		**6**	**2**	**1**	**5**	**5**	**12**	**31**	

*Allocated by database http://www.mobovis.org/; C = Cattle strain Identification number.

Abbreviations used for districts (*n *= 31): LSK = Lusaka; MZK = Mazabuka; CHM = Choma; MBW = Mumbwa; MZE = Monze; NMA = Namwala.

**Figure 2:**
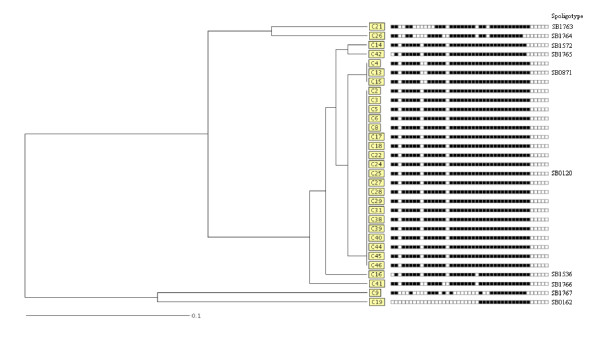
**Relationship of spoligotypes of *M. bovis *isolates from Zambian cattle**. The presented patterns were generated using the band-based dice coefficient and clustering determined by the unweighted pair group algorithm with arithmetic averages (UPMGA) method. Designation of spacers from left to right is 1 to 43. Numbers on the right represent spoligotypes described in the international database http://www.mbovis.org.

Ten different spoligotypes were distinguished (Table 1 and Figure 2). Twenty-seven isolates belonged to one cluster with more than 95% similarity (Figure 2); they all have spacers 2, 4–8, 11–14, 17–23 and 25–37. Inside the cluster, one predominant spoligotype was found in 20 (64.5%) of the isolates tested. It was found in animals originating from 5 of the 6 study districts. The second most prevalent spoligotype was found in isolates from three districts; C4 from Namwala, C13 from Choma and C15 from Mumbwa (Table 1 and Figure 2). Three isolates in the cluster, C16 and C42 from Namwala and C14 from Lusaka are closely related to each other with only spacer 1, 24 and 38 being different (Figure 2).

Four isolates, C21, C26, C9 and C19, showed a low degree of similarity with the other 27 isolates. Isolate C9 from Monze district and C19 from Namwala are clearly distinct from the rest; C19 is lacking all the spacers from 1 to 24 (Figure 2).

In terms of geographic variability, Namwala district had a total of 7 spoligotypes of which 5 isolates (C19, C26, C42, C16 and C41) were only present in the Namwala district (Table 1).

Based on the global spoligotype patterns diversity provided by the international data base on spoligotyping, http://www.mbovis.org, 83.9% of the isolates have spoligo patterns that have been described in other countries (Table 2). The predominant spoligotype, widely dispersed geographically (Table 1 &2), was found in the international data base to have a pattern with a spoligotype number SB0120 with the corresponding hexacode of 6F-5F-5F-7F-FF-60. Five out of the six study districts had this predominant spoligotype, and Namwala district accounted for 30% of spoligotype SB0120. The second most predominant spoligotype had a pattern named SB0871 with a corresponding hexacode of 6F-4F-5F-7F-FF-60. Isolates C14 was named SB1572 with a hexacode number of 6F-5F-5F-7F-FF-40, isolate C16 was SB1536 with a hexacode number of 2F-5F-5F-6F-FF-60 and isolate C19 was SB0162 with a hexacode number of 00-00-00-0F-FF-60. The distribution of these spoligotypes on the international data base is shown in Table 2.

**Table 2:** Major Spoligotypes in Zambia

Spoligotype^1^	Shared type^2^	Geographical distribution
Sp1	SB0120	France, Belgium, Brazil, South Africa, Sri Lanka, Iran, The Netherlands, Spain, China, Japan, Portugal, Russia, Denmark, Zambia
Sp2	SB0871	France
Sp3	SB1763*	Zambia
Sp4	SB1764*	Zambia
Sp5	SB1572	Italy
Sp6	SB1765*	Zambia
Sp7	SB1536	Italy
Sp8	SB1766*	Zambia
Sp9	SB1767*	Zambia
Sp10	SB0162	Belgium

^1 ^Arbitrary spoligotype designation

^2 ^Shared type, designation of the spoligotype in the World Spoligotype Database.

*New Spoligotype assigned by http://www.mbovis.org

Five individually occurring isolates (16.1%) displayed new spoligo patterns that have not yet been described on the international spoligotyping data base (Figure 2 and Table 2). These isolates originated from Namwala district (isolate C26, 42 and C41); from Mumbwa (isolate C21); and from Monze (isolate C9) (Table 1 and Figure 2). These new patterns were allotted new spoligo numbers as SB1763 (hex code 66-03-5F-6D-FF-60), SB1764 (hex code 60-0F-1F-6C-FF-00), SB1765 (hex code 2F-5F-5F-7F-FF-40), SB1766 (hex code 6F-4F-1F-6F-FF-60) and SB1767 (hex code 62-0E-50-09-FF-40) by http://www.mbovis.org Table 2.

The technique showed a good discrimination power; Hunter Gaston Discriminatory Index (HGDI = 0.98) (Table 1 and Figure 2.).

## Discussion

Our results do not agree with what has been found in other parts of Africa [21,22], where more than 40% of the animals with tuberculous lesions had Non-tuberculous Mycobacteria (NTM). In this study, only two animals had mycobacteria other than *M. bovis*. However, our findings tie up with a similar study conducted in Algeria [23]. Whereas excluding the differences in bacterial species as accounting for these observations [23], strain isolation has been found to be dependant on the specific type of media used [24]. The usage of specific culture media such as Stonebrink has been shown to increase the recovery and discrimination of strains on culture [25,26]. However, in this study, the use of Lowenstein Jensen media with pyruvate enrichment may to a lesser extent account for the low numbers of NTMs although not conclusively established. This study is the first of its kind in Zambia to describe the molecular typing of *M.bovis *isolates from indigenous cattle breeds originating from high prevalence settings.

Characterization of *M. bovis *strains based on different geographical locations by districts or region is pivotal in understanding the molecular epidemiology of BTB [21,23,27]. It further helps in understanding the dynamics of disease dispersion which are difficult to appreciate through traditional epidemiological investigative tools. However, through the use of modern molecular epidemiological tools such as spoligotyping, we have been able to demonstrate the presence as well as the specific existing strains of *Mycobacterium bovis *in Zambian cattle. The technique has shade more light on the strain diversity, distribution and relatedness within Zambia and globally. Two dominant spoligotypes were identified representing the majority of isolates analyzed. These findings intimate a degree of homogeneity among *M. bovis *isolates in Zambia. However, when distinguishing between unrelated strains through the application of the Hunter-Gaston Discriminatory Index [28,29], the spoligotyping technique in this particular case was found to have a good discrimination power. The index indicated that 98% of the strains had an equal chance of having different spoligo patterns if randomly sampled.

Of the 31 *M. bovis *isolates that yielded interpretable spoligotypes, 10 different patterns were detected. Based on the global spoligotype patterns diversity provided by the international data base on spoligotyping, http://www.mbovis.org, 83.9% of the isolates have been described. The predominant spoligotype that was widely dispersed geographically was found on the international data base to have a pattern with a spoligotype number SB0120. This spoligotype is similar to the spoligotype of the vaccine strain BCG type, and previously described in France, Belgium, South Africa, The Netherlands, Sri Lanka, Spain, Japan, Portugal, Russia, Iran, Denmark, China and Brazil http://www.mbovis.org[30]. The second most predominant spoligotype had a pattern previously numbered SB0871 and has been described from France. These predominant patterns, SB0120 and SB0871, differ only by a single spacer (spacer 10). The most common spoligotype, SB0120, has a considerable degree of geographical dispersion in Zambia, being detected in 5 out of the 6 districts, and has further been shown to be common in other countries including continental Europe [31,32]. This finding of strains from Europe may suggest the introduction of the disease by early European settlers to Africa, a finding that has been highlighted by different workers [17,23,27]. The finding of SB0120 in South Africa strongly infers to this probability, when tracing the early migration routes of colonial settlers to Zambia. In our current study, 16.1% (5/31) of the isolates had spoligotypes that were unique to Zambia. These spoligotypes were newly described considering that no previous information of their registration on the international data base existed. These patterns (SB1763–1767) reveal deletion events that could have lead to new spoligotype patterns evolving, as was the case in Portugal [30]. However, more detailed studies need to be conducted to fully ascertain this assertion.

The sharing of grazing land in the Kafue Basin in Zambia between cattle and Kafue lechwe antelopes (*Kobus leche Kafuensis*), considered as wildlife biological reservoir hosts for BTB, might explain the high prevalence levels found in this setting [3,4,6,33]. Underlying factors in sustaining the infectious agent depend on the temporal and spatial distribution between the source of infection and the susceptible animals, which also are a function of the duration of interaction between the agent, the susceptible host and its environment [34]. The underlying factors for BTB transmission between the Lechwe antelopes and cattle are reported to be optimal in the Kafue Basin [3,6], although further investigations at molecular level will be necessary to elucidate this relationship.

The tracing of livestock movement patterns from their areas of origin to major abattoirs is important in understanding possible disease dispersion patterns. Cattle traders trek for days from areas within and around the Kafue Basin to abattoirs in the nearby districts[3]. In our study, we observed that identical and closely related strains were also found in other districts. These findings suggest the sharing of strains between districts, a finding which is important when determining BTB localization or spread.

Namwala district (the only district right in the Kafue Basin) [8] was found to have more isolates than any other district. The practice of allowing trekking animals to spend one or more nights in different kraals during the journey to abattoirs may partially be responsible for the dissemination of the infectious organisms. This pattern of animal movements may to a greater extent be responsible to the observed dispersion of spoligotype patterns suggestively, based on our results from the Namwala district zones to other surrounding districts. However, these results need to be interpreted with caution considering limitations related to the survey period and sample size related to limited resources and time constraints. This makes the results a bit difficult to interpret when inferring to the entire region given the representativeness of the sample size. In addition, the spoligotyping technique has weaknesses in that it has a low discriminatory power [29] which may result in low specificity of some patterns with a possibility of grouping strains that might not be identical when typed by other methods. However, the results obtained in this study give an indication of *M. bovis *strains in cattle with an insight in the likely role that cattle movements have on the dissemination of the disease.

These findings are useful in assisting policy makers when considering the formulation of control measures for BTB in Zambian cattle. It is important for policy makers to base their control polices on researched scientific evidence. This study has highlighted that unrestricted cattle movements to abattoirs may play a major contributory role in the dissemination of BTB. Thus policy makers should consider building abattoirs in all areas of high cattle production and further formulate a policy that will stop cattle movements "on the hoof" which will compel cattle owners to use trucks when transporting animals to abattoirs.

## Conclusion

This study has described spoligotypes of *M.bovis *in Zambian cattle for the first time, and has identified five spoligotypes that are specific to the country. The observation of an overlap in the spoligotype pattern SB0120 in 5 of the 6 districts suggests a possible common source of infection.

## Methods

### Specimen source areas

The southern parts of Zambia are endowed with flood plains, which have suitable grazing grounds for both wild and domesticated animals. One such flood plain is the Kafue Basin which is surrounded by seven major districts (like counties) with a lot of sub districts/small towns within the major ones, supplying cattle to the main abattoirs in Lusaka, the capital city (Figure 1). More than over two-thirds of the Zambian cattle population which number about 2,500,000 animals are found in the southern region [8] with the traditional livestock sector accounting for more than 80% of the national population. The traditional sector consists of four distinct indigenous cattle breeds; the Agoni, a shorthorn Zebu (*Bos indicus*) breed from eastern Zambia; Tonga and Baila, Sanga breeds (cross breeds of *Bos indicus *and *Bos taurus*) from southern Zambia and the Barotse cattle, a Sanga breed from western Zambia. Based on epidemiological studies conducted on BTB in cattle[1,4], animals from the southern region were followed along the slaughter line and screened for any visible tuberculous lesions from March to June 2004.

### Sampling

Slaughtered animals were followed along the examination line and examined for gross lesions according to the standard post mortem examination procedures by [35]. Organs and tissues with suspected TB lesions were collected after detailed postmortem examination of the entire carcass. Demographic data of area of origin, sex, age type of organ or tissue was recorded as well as the type of gross pathological postmortem disposition. These specimens were placed in sterile self zipping histopathological bags, placed into a cooler box with ice packs before transport to the laboratories where they were stored in a standard fridge (within four days) during processing for culturing or kept at -20°C if not processed within four days.

### Decontamination and Culturing

All the BTB suspect tissues and organs were decontaminated in the Biohazard Safety Cabinet in a Bio-safety Level 2 laboratory. Fat was trimmed off from the suspect material of which some 3 to 6 g was measured in a sterile mortar to which 10 ml of 4% sodium hydroxide was added. An equal amount of sterile sand was added to the contents in the mortar and ground. The products were then transferred to McCartney bottles and centrifuged at low speed of 3000 rpm for 10 minutes. Thereafter, the supernatant fluid was decanted off and 20 mls of sterile water was added to the sediment and mixed vigorously by vortexing to a uniform homogenate. The contents were again centrifuged at low speed of 3000 rpm for 20 minutes and the supernatant fluid was decanted. The sediments of these decontaminated homogenates were inoculated in duplicate Lowenstein-Jensen media slants supplemented with 0.4% sodium pyruvate to enhance the isolation of *M. bovis *and incubated aerobically at 37°C for 8 weeks. The resulting cultures were tentatively identified as probable *Mycobacterium tuberculosis-*complex by their slow growth and colony morphology. Purity and acid-fastness of the colonies were checked by Zhiel Neelsen staining.

### Preparation of lysates and molecular typing of isolates

Cell lysates were prepared by suspending a loop full of bacterial colony in 250 μl of 1× TE buffer (10 m*M *Tris/HCl, pH8.0 and 1 m*M *EDTA in distilled water) in an Eppendorf tube. Bacterial cells were heat killed by incubation at 80°C for 1 hour in a temperature controlled water bath. After centrifuging the cells at 13000 rpm for 2 minutes, the supernatant was discarded and the pellet resuspended in 500 μl of 150 m*M *sodium chloride. This step was repeated twice. Finally, the supernatant was discarded and the pellet resuspended in 25 μl 1× TE buffer. These suspensions were used for spoligotyping as previously described [15]. Four microliters (4 μl) of the denatured bacterial suspension from each sample was used for amplification of the direct-repeat (DR) region. The labelled amplicons were used as probes for hybridization with a set of 43 known oligonucleotide spacer sequences. The H37Rv *M. tuberculosis*, and *M. bovis *BCG P3 strains, and purified water were included in each experiment as positive and negative controls, respectively. Bound PCR fragments were detected with a streptavidinhorseradish peroxidase-enhanced conjugate and an enhanced chemiluminescence (ECL) system, followed by exposure to ECL hyperfilms (Amersham Pharmacia-Biotech, Roosendael, The Netherlands). The expected patterns of the positive controls were observed and no reagent contamination was detected in all the negative controls. The spoligotypes were compared using the band-based Dice coefficient and clustering determined by the unweighted pair group algorithm with arithmetic averages (UPMGA) method, using the MIRU-VNTR *plus *software[36]

### Calculating the Discriminatory power

Hunter-Gaston Discriminatory Index (HGDI) equation was used for the calculation of the discriminatory power for the set of strains that were used in this study [28,29]. The equation used read as follows:

Where *D *is the Index of discriminatory power, *s *is the total number of the types to be described *nj *is the number of strains in the population which are indistinguishable from the *j*^*th *^strain, and *N *is the total number of strains in the sample population. The discriminatory power was calculated based on the consideration of an isolate per particular spoligotype in the study using the above formula derived from elementary probability theory.

## Authors' contributions

MM contributed to the design, data collection, laboratory experiments, and analysis of data and drafting of the manuscript. LR contributed to the design, supervision of molecular typing, drafting and writing of manuscript. ICS contributed to carrying out molecular genetic studies, supervision of the work, drafting and reviewing of the manuscript. JBM contributed to the collection of field data in and drafting of the manuscript. MT contributed to supervision of the project, acquisition of parts of the funds and writing of the manuscript. ES contributed to the writing of manuscript. BD contributed to conception and design, data analysis and the writing of manuscript. All authors have read and approved the final manuscript.
